# Supplementary material for: A critical evaluation for validation of composite and unidimensional postoperative pain scales in horses
Source: PLoS One. 2021 Aug 5;16(8):e0255618. doi: 10.1371/journal.pone.0255618 (PMC8341545; doi:10.1371/journal.pone.0255618)
Supplement: S1 Table — (PDF) [file pone.0255618.s001.pdf]

S1 Table. Demographic data including sex, breed, age, weight, procedure and peri-operative analgesia, and institution of each horse included in this validation study.

Horse	Sex	Breed	Age (years)	Weight (kg)	Procedure	Peri-operative analgesia	Institution
1	Gelding	Thoroughbred	4	544	Osteosynthesis	Phenylbutazone PO	University of Pennsylvania
2	Stallion	Standardbred	3	500	Osteosynthesis	Phenylbutazone PO	University of Pennsylvania
3	Gelding	Warmblood	16	581	Desmotomy	Phenylbutazone PO	University of Pennsylvania
4	Mare	Standardbred	3	385	Arthroscopy	Phenylbutazone PO	University of Pennsylvania
5	Gelding	Thoroughbred	2	477	Arthroscopy & condylar fracture repair	Phenylbutazone PO	University of Pennsylvania
6	Mare	Thoroughbred	3	480	Condylar fracture repair	Phenylbutazone PO	University of Pennsylvania
7	Gelding	Thoroughbred	6	453	LF fetlock arthrodesis	Phenylbutazone PO	University of Pennsylvania
8	Mare	Thoroughbred	4	540	LF fetlock arthroscopy	Phenylbutazone PO	University of Pennsylvania
9	Gelding	Thoroughbred	8	485	LH fetlock arthroscopy	Phenylbutazone PO	University of Pennsylvania
10	Gelding	Belgian Warmblood	15	648	R stifle arthroscopy	Phenylbutazone PO	University of Pennsylvania
11	Stallion	Thoroughbred	2	486	Osteosynthesis	Phenylbutazone PO	University of Pennsylvania
12	Gelding	Warmblood	8	603	LF tenoscopy	Phenylbutazone PO	University of Pennsylvania
13	Stallion	Thoroughbred	2	485	LF fetlock arthroscopy	Phenylbutazone PO	University of Pennsylvania
14	Gelding	Warmblood	16	640	L stifle arthroscopy	Phenylbutazone PO	University of Pennsylvania
15	Gelding	Thoroughbred	5	476	L laryngoplasty & laser cordectomy	Phenylbutazone PO	University of Pennsylvania
16	Gelding	Warmblood	9	662	RH fasciotomy neurectomy	Phenylbutazone PO	University of Pennsylvania
17	Gelding	Warmblood	1	444	L hock arthroscopy	Phenylbutazone PO	University of Pennsylvania
18	Gelding	Thoroughbred	8	600	Splint fracture bone removal	Phenylbutazone PO	University of Pennsylvania
19	Gelding	Welsh Cross	17	480	LF & RF MRI	None	University of Pennsylvania
20	Gelding	Thoroughbred	23	456	Phallectomy	Phenylbutazone PO	University of Pennsylvania
21	Gelding	Thoroughbred	4	589	L stifle arthroscopy	Phenylbutazone PO	University of Pennsylvania

22	Stallion	Thoroughbred	2	571	LF pastern arthrodesis	Phenylbutazone IV, epidural morphine	University of Pennsylvania
23	Gelding	Thoroughbred	2	449	L carpal arthroscopy	Phenylbutazone PO	University of Pennsylvania
24	Mare	Warmblood	17	580	LF splint bone removal	Phenylbutazone PO	University of Pennsylvania
25	Mare	Thoroughbred	3	500	LF tendon lavage	Phenylbutazone IV	University of Pennsylvania
26	Gelding	Warmblood	25	555	L hock arthroscopy	Phenylbutazone PO	University of Pennsylvania
27	Mare	Arabian	17	421	R elbow plate removal	Phenylbutazone PO	University of Pennsylvania
28	Stallion	Standardbred	1	430	MCIII screw placement	Phenylbutazone PO	University of Pennsylvania
29	Mare	Warmblood Cross	7	471	R stifle nodulectomy	Phenylbutazone PO	University of Pennsylvania
30	Mare	Warmblood	8	462	Superior check ligament desmotomy	Phenylbutazone PO	University of Pennsylvania
31	Gelding	Warmblood	7	555	LH tenoscopy	Phenylbutazone PO	University of Pennsylvania
32	Stallion	Thoroughbred	2	467	L carpal arthroscopy	Phenylbutazone PO	University of Pennsylvania
33	Gelding	Arabian	5	280	LF fetlock arthroscopy	Flunixin meglumine IV	Unesp
36	Mare	Mixed breed	7	303	LF fetlock arthroscopy	Flunixin meglumine IV	Unesp
37	Stallion	Mixed breed	15	411	Tooth extraction & sinuscopy	Phenylbutazone PO	University of Ghent
38	Gelding	Royal Dutch Sport Horse	5	546	Hock arthroscopy	Phenylbutazone PO	University of Ghent
39	Gelding	N/A	16	522	Thyroidectomy	Phenylbutazone PO	University of Ghent
40	Stallion	Warmblood	5	497	Castration	Phenylbutazone PO	University of Ghent
41	Mare	Warmblood	6	540	Sarcoid excisions	Phenylbutazone PO	University of Ghent
42	Stallion	N/A	24	370	Enucleation	Phenylbutazone PO	University of Ghent
43	Stallion	Mangalarga	7	415	Castration	Flunixin meglumine IV	Unesp
44	Stallion	Mangalarga	3	348	Castration	Flunixin meglumine IV	Unesp

IV: intravenous; L: left; LF: left front; LH: left hind; N/A: information not available; R: right; MCIII: third metacarpal bone; RH: right hind; PO: oral.
