# Supplementary material for: A critical evaluation for validation of composite and unidimensional postoperative pain scales in horses
Source: PLoS One. 2021 Aug 5;16(8):e0255618. doi: 10.1371/journal.pone.0255618 (PMC8341545; doi:10.1371/journal.pone.0255618)
Supplement: S2 Table — (PDF) [file pone.0255618.s002.pdf]

S2 Table. Anesthetic and analgesic drug protocols

Horse	Premedication	Induction	Perianesthetic medication	Maintenance	Post-Surgery	Remarks	Period
1	Acepromazine, Detomidine, Xylazine	EGG, Ketamine	Phenylbutazone, Xylazine	Isoflurane			Morning
2	Xylazine, Butorfanol, Detomidine	Midazolam, Ketamine	Phenylbutazone, Bupivacaine block, Acepromazine	Isoflurane	Xylazine		Afternoon
3	Xylazine, Midazolam, Ketamine		Phenylbutazone	Isoflurane	Xylazine		Afternoon
4	XylazCTine	Midazolam, Ketamine	Phenylbutazone, Xylazine	Isoflurane		Xylazine	Morning
6	Acepromazine, Detomidine, Butorphanol, Xylazine	Midazolam, Ketamine	Dexmedetomidine, Butorphanol	Isoflurane		Pool recovery	Morning
5	Xylazine, Acepromazine, Detomidine, Phenylbutazone	Midazolam, Ketamine	Acepromazine	Isoflurane, CRI Xylazine	Telazoline	Standing CT	Afternoon
7		Midazolam, Ketamine	Phenylbutazone	Isoflurane, CRI Xylazine	Xylazine, Acepromazine	Pool recovery	Afternoon
8	Acepromazine, Detomidine, Xylazine, Phenylbutazone	Midazolam, Ketamine	Detomidine, Dexmedetomidine, Local Block Bupivacaine, Acepromazine		Telazoline		Morning

9	Xylazine, Phenylbutazone	Midazolam, Ketamine	Butorphanol, Acepromazine		Detomidine, Acepromazine	Sedation with Detomidine	Morning
10	Acepromazine, Detomidine, Xylazine, Butorfanol, Phenylbutazone	Midazolam, Ketamine	Butorphanol	Isoflurane, CRI Xylazine		Flumazenil	Afternoon
11	Acepromazine, Detomidine, Xylazine, Phenylbutazone	Midazolam, Ketamine	Detomidine, Xylazine	Isoflurane		Xylazine	Afternoon
12	Acepromazine, Detomidine, Xylazine	Midazolam, Ketamine	Butorphanol, Phenylbutazone, Bupivacaine (Block), Xylazine	Isoflurane, CRI Xylazine	Xylazine	Sedation with Detomidine	Morning
13	Acepromazine, Detomidine, Xylazine, Phenylbutazone	Midazolam, Ketamine	Dexmedetomidine, Acepromazine	Isoflurane		Pre-surgery Phenylbutazone PO	Morning
14	Acepromazine, Detomidine, Xylazine	ECG, Ketamine	Butorphanol, Ketamine, Xylazine	Isoflurane, CRI Xylazine	Xylazine, Acepromazine, Tolazoline	Increased creatinine, Phenylbutazone PO Pre-surgery	Morning
15	Acepromazine, Detomidine, Xylazine	Midazolam, Ketamine	Phenylbutazone, Dexametasona, Butorphanol, Detomidine	Isoflurane	Xylazine	Dexamethasone	Morning
16	Xylazine, Phenylbutazone	Midazolam, Ketamine	Butorphanol, Acepromazine	Isoflurane, Bupivacaine Splash	Xylazine		Morning

17	Acepromazine, Detomidine, Xylazine	Midazolam, Ketamine	Xylazine, Phenylbutazone	Isoflurane			Morning
18	Acepromazine, Detomidine, Xylazine	Midazolam, Ketamine	Butorphanol, Xylazine, Acepromazine, Detomidine	Isoflurane		Phenylbutazone PO pre-surgery	Morning
19	Xylazine	Midazolam, Ketamine	Phenylbutazone, Acepromazine	Isoflurane	Xylazine	Sedation with xylazine	Morning
20	Xylazine, Butorphanol, Xylazine	Midazolam, Ketamine	Ketamine, Acepromazine, Lido	Desflurane, CRI Lidocaine	Xylazine	Phenylbutazone PO	Morning
21	Acepromazine, Detomidine, Xylazine	Midazolam, Ketamine	Phenylbutazone, Xylazine, Intraarticular Bupivacaine, Butorphanol, Dexmedetomidine			Xylazine, Detomidine, Phenylbutazone (PO). Rescue analgesia with morphine/xylazine via epidural Catheter	Morning
22	Acepromazine, Xylazine, Phenylbutazone	Midazolam, Ketamine	Dexmedetomidine,	Desflurane, Local Bupivacaine	Flumazenil, Tolazoline, Xylazine		Morning
23	Xylazine	Midazolam, Ketamine	Phenylbutazone, Butorphanol Bolus, Acepromazine	Isoflurane	Xylazine		Morning
24	Phenylbutazone, Acepromazine, Xylazine	Midazolam, Ketamine	Butorphanol	Isoflurane	Xylazine		Afternoon

25	Xylazine	Midazolam, Ketamine	Butorphanol, Xylazine	Desflurane, Dexmedetomidine	Flumazenil	Patient lying on M3, Continuous rate infusion of Gabapentin, Phenylbutazone IV	Afternoon
26	Acepromazine, Detomidine, Phenylbutazone	Xylazine,Ketamine, Midazolam	Butorphanol, Acepromazine	Isoflurane, CRI Xylazine			Morning
27	Acepromazine, Xylazine, Butorphanol, Phenylbutazone	Midazolam, Ketamine	Butorphanol, Dexmedetomidine	Isoflurane	Flumazenil		Morning
28	Acepromazine, Detomidine, Phenylbutazone	Xylazine, Midazolam, Ketamine	Butorphanol	CRI Xylazine, Isoflurane			Morning
29	Xylazine	Midazolam, Ketamine	Phenylbutazone, Butorphanol Bolus	Isoflurane	Acepromazine, Xylazine		Morning
30	Xylazine, Butorphanol	Midazolam, Ketamine	Phenylbutazone, Butorphanol Bolus	Isoflurane	Acepromazine, Telazoline	Detomidine 5h before M0	Morning
31	Acepromazine, Detomidine	Ketamine, Xylazine	Phenylbutazone, Butorphanol Bolus	Isoflurane	Romifidine	MRI	Morning
32	Xylazine	Midazolam, Ketamine	Phenylbutazone	Isoflurane	Xylazine, Acepromazine	M0 6h before procedure	Morning
33	Acepromazine, Xylazine	Diazepam, Ketamine		Isoflurane, CRI Lidocaine, Block Lidocaine	Flunixin meglumine		Afternoon
36	Acepromazine, Xylazine	Diazepam, Ketamine		Isoflurane, CRI Lidocaine, Block Lidocaine	Xylazine, Flunixin meglumine		Afternoon

37	Detomidine			CRI Detomidine, Mepivacaina Local			Morning
38	Morphine, Romifidine	Diazepam, Ketamine		CRI Romifidine, Isofluraneflurane			Morning
39	Morphine, Romifidine			CRI Romifidine			Morning
40	Morphine, Romifidine			CRI Romifidine			Morning
41	Morphine, Romifidine			CRI Romifidine			Morning
42	Detomidine, Morphine			CRI Romifidine, Local Mepivacaine			Morning
43	Acepromazine, Xylazine	Diazepam, Ketamine		Isoflurane, Lidocaine Intratesticular Block	Ketamine, Flunixin meglumine		Morning
44	Acepromazine, Xylazine	Ketamine, Diazepam		Isoflurane, Intratesticular Lidocaine	Flunixin meglumine	Developed colic after M3	Morning

CRI – constant rate infusion; CT – computed tomography; IV – intravenous; MRI – magnetic resonance image; PO – oral.
