# Supplementary material for: A critical evaluation for validation of composite and unidimensional postoperative pain scales in horses
Source: PLoS One. 2021 Aug 5;16(8):e0255618. doi: 10.1371/journal.pone.0255618 (PMC8341545; doi:10.1371/journal.pone.0255618)
Supplement: S3 Table — (PDF) [file pone.0255618.s003.pdf]

S3 Table. Repeatability of the UHAPS, CPS and unidimensional scales to assess perioperative pain in horses

	Intra-observer reliability [(kappa* or intraclass correlation** coefficients (confidence interval 95%)]					
	**Reference Evaluator**	**Lead Investigator**	**Anesthesiologist**	**Veterinary Technician**	**Equine internist**	**Veterinary Student**
UHAPS						
Positioning in the stall*	0.93 (0.87-0.98)	0.91 (0.86-0.95)	0.73 (0.61-0.85)	0.78 (0.67-0.88)	0.65 (0.53-0.76)	0.73 (0.63-0.84)
Locomotion*	0.90 (0.84-0.97)	0.90 (0.84-0.97)	0.80 (0.67-0.93)	0.58 (0.46-0.70)	0.53 (0.38-0.68)	0.50 (0.25-0.74)
Locomotion when led by the evaluator*	0.92 (0.86-0.98)	0.92 (0.86-0.98)	0.77 (0.61-0.93)	0.67 (0.54-0.79)	0.69 (0.59-0.79)	0.73 (0.62-0.85)
Response to palpation of the painful area*	0.91 (0.84-0.98)	0.88 (0.82-0.94)	0.68 (0.51-0.84)	0.69 (0.60-0.79)	0.82 (0.74-0.90)	0.80 (0.70-0.89)
Looking at the flank*	0.96 (0.88-1.00)	0.66 (0.04-1.00)	0.49 (-0.11-1.00)	0.66 (0.05-1.00)	0.42 (0.09-0.76)	0.66 (0.05-1.00)
Kicking at the abdomen*	1.00 (1.00-1.00)	1.00 (1.00-1.00)	0.66 (0.05-1.00)	0.28 (0.56-0.80)	0.58 (0.34-0.83)	0.80 (0.41-1.00)
Lifting hind limbs*	0.89 (1.00-1.00)	0.80 (0.65-0.94)	0.78 (0.63-0.94)	0.32 (0.15-0.49)	0.56 (0.41-0.70)	0.49 (0.23-0.75)
Head movements*	0.89 (0.78-1.00)	0.87 (0.76-0.99)	0.80 (0.65-0.95)	0.50 (0.30-0.70)	0.53 (0.39-0.67)	0.67 (0.40-0.95)
Pawing on the floor*	0.77 (0.54-0.99)	0.57 (0.28-0.85)	0.33 (0.16-0.81)	0.60 (0.28-0.92)	0.64 (0.41-0.87)	0.76 (0.50-1.00)
**UHAPS total score****	**0.96 (0.94-0.97)**	**0.95 (0.93-0.96)**	**0.82 (0.76-0.87)**	**0.80 (0.73-0.85)**	**0.77 (0.70-0.83)**	**0.79 (0.73-0.84)**
CPS						
Appearance*	0.85 (0.76-0.94)	0.91 (0.84-0.97)	0.66 (0.43-0.90)	0.64 (0.54-0.75)	0.62 (0.51-0.74)	0.63 (0.52-0.75)
Kicking at abdomen*	0.66 (0.16-1.00)	1.00 (1.00-1.00)	0.50 (-0.1-1.00)	0.28 (-0.23-0.80)	0.63 (0.34-0.93)	0.80 (0.40-1.00)
Pawing on the floor*	1.00 (1.00 -1.00)	0.96 (0.91-1.00)	0.08 (-0.13-0.31)	0.46 (0.22-0.71)	0.64 (0.48-0.80)	0.73 (0.55-0.90)
Posture*	0.91 (0.85-0.97)	0.91 (0.84-0.97)	0.83 (0.71-0.94)	0.73 (0.63-0.82)	0.74 (0.62-0.85)	0.79 (0.70-0.88)
Head movement*	0.91 (0.83-0.99)	0.77 (0.66-0.88)	0.72 (0.50-0.94)	0.57 (0.35-0.79)	0.54 (0.36-0.85)	0.81 (0.61-1.00)
Appetite*	0.91 (0.86-0.97)	0.92 (0.87-0.97)	0.79 (0.70-0.89)	0.74 (0.64-0.83)	0.73 (0.63-0.83)	0.82 (0.74-0.89)
Response to observer*	0.97 (0.90-1.00)	0.55 (0.16-0.93)	0.66 (0.17-1.00)	0.71 (0.19-1.00)	0.66 (0.51-0.82)	0.93 (0.78-1.00)
Response to palpation of the painful area*	0.86 (0.79-0.93)	0.86 (0.79-0.93)	0.70 (0.56-0.84)	0.71 (0.61-0.82)	0.83 (0.77-0.89)	0.84 (0.78-0.90)
**CPS total score****	**0.95 (0.93-0.96)**	**0.94 (0.91-0.95)**	**0.83 (0.77-0.87)**	**0.85 (0.80-0.89)**	**0.80 (0.74-0.85)**	**0.83 (0.78-0.87)**

Indication of rescue analgesia***	0.92 (0.84-1.00)	0.90 (0.81-0.98)	0.74 (0.55-0.92)	0.66 (0.52-0.81)	0.67 (0.55-0.78)	0.58 (0.44-0.72)
**Simple descriptive scale***	0.95 (0.93-0.98)	0.94 (0.92-0.97)	0.87 (0.81-0.92)	0.83 (0.76-0.89)	0.84 (0.79-0.90)	0.72 (0.59-0.86)
**Simple numeric scale***	0.98 (0.97-0.99)	0.98 (0.97-0.99)	0.94 (0.91-0.96)	0.82 (0.72-0.93)	0.87 (0.82-0.93)	0.70 (0.58-0.83)
**Visual analog scale****	0.98 (0.97-0.99)	0.98 (0.97-0.98)	0.94 (0.92-0.96)	0.86 (0.82-0.90)	0.88 (0.83-0.91)	0.75 (0.67-0.81)

UHAPS- Unesp-Botucatu horse acute pain scale; CPS - Composite Pain Scale. Interpretation of Kappa coefficient results; kw 0.81-1.0 very good; 0.61-0.80 good; 0.41-0.60 moderate; 0.21-0.4 reasonable; < 0.2 poor [37,38] .* Intraclass correlation coefficient (confidence interval of 95%).
