# Supplementary material for: A critical evaluation for validation of composite and unidimensional postoperative pain scales in horses
Source: PLoS One. 2021 Aug 5;16(8):e0255618. doi: 10.1371/journal.pone.0255618 (PMC8341545; doi:10.1371/journal.pone.0255618)
Supplement: S4 Table — (PDF) [file pone.0255618.s004.pdf]

S4 Table. Reproducibility of the UHAPS, CPS and unidimensional scales to assess perioperative pain in horses.

	Inter-observer reliability [(kappa* or intraclass correlation** coefficients (confidence interval 95%)] Reference evaluator *versus*				
**UHAPS**	**Lead investigator**	**Anesthesiologist**	**Veterinary technician**	**Equine internist**	**Veterinary student**
Positioning in the stall*	0.54 (0.41-0.68)	0.53 (0.40-0.67)	0.65 (0.55-0.75)	0.41 (0.27-0.55)	0.60 (0.48-0.72)
Locomotion*	0.90 (0.84-0.97)	0.38 (0.24-0.51)	0.34 (0.23-0.45)	0.28 (0.18-0.38)	0.06 (-0.03-0.14)
Locomotion when led by the evaluator*	0.92 (0.86-0.98)	0.59 (0.46-0.72)	0.50 (0.37-0.64)	0.46 (0.33-0.59)	0.57 (0.44-0.70)
Response to palpation of the painful area*	0.52 (0.40-0.64)	0.68 (0.58-0.78)	0.56 (0.43-0.69)	0.38 (0.25-0.52)	0.58 (0.46-0.69)
Looking at the flank*	0.25 (-0.03-0.53)	0.54 (0.26-0.81)	0.13 (-0.10-0.37)	0.25 (-0.03-0.53)	0.13 (-0.10-0.37)
Kicking at the abdomen*	-0.01 (-0.02-0)	0.35 (-0.01 – 0.71)	-0.01 (-0.02-0.00)	0.66 (0.05-1.00)	0.39 (-0.16-0.94)
Lifting hind limbs*	0.89 (0.79-1.00)	0.37 (0.20 -0.54)	0.27 (0.08-0.47)	0.47 (0.27-0.68)	0.41 (0.19-0.63)
Head movements*	0.87 (0.76-0.99)	0.35 (0.17 – 0.52)	0.15 (0.-0.31)	0.27 (0.12-0.42)	0.27 (0.12-0.43)
Pawing on the floor*	0.77 (0.54-0.99)	0.34 (0.07-0.61)	0.47 (0.16-0.79)	0.21 (-0.14-0.57)	0.65 (0.37-0.94)
**Total score UHAPS****	**0.83 (0.78-0.87)**	**0.73 (0.65-0.79)**	**0.63 (0.53-0.71)**	**0.40 (0.26-0.52)**	**0.49 (0.36-0.59)**
**CPS**					
Appearance*	0.66 (0.55-0.77)	0.27 (0.16-0.39)	0.43 (0.29-0.57)	0.13 (0.01-0.26)	0.00 (-0.12-0.13)
Kicking at the abdomen*	0.22 (-0.18- 0.61)	0.17 (-0.04-0.39)	0.22 (-0.18-0.61)	0.22 (-0.18-0.61)	0.35 (-0.08-0.78)
Pawing on the floor*	0.94 (0.90-0.98)	0.51 (0.20-0.82)	0.39 (0.12-0.65)	0.02 (-0.6-0.10)	0.52 (0.21-0.83)

Posture*	1.00 (1.00-1.00)	0.62 (0.48-0.76)	0.55 (0.55-076)	0.60 (0.48-0.72)	0.46 (0.29-0.62)
Head movement*	0.91 (0.84-0.98)	0.50 (0.32-0.68)	0.29 (0.10-0.48)	0.25 (0.11-0.39)	0.35 (0.15-0.55)
Appetite*	1.00 (1.00-1.00)	0.66 (0.55 – 0.76)	0.62 (0.51-0.72)	0.49 (0.36-0.61)	0.49 (0.39-0.58)
Response to observer*	0.07 (-12-0.26)	0.10 (-0.05 -0.25)	0.47 (-0.02-0.95)	0.39 (-0.13-0.92)	0.40 (-0.06-0.85)
Response to palpation of the painful area*	1.00 (1.00-1.00)	0.70 (0.61 – 0.79)	0.49 (0.34-0.64)	0.40 (0.25-0.55)	0.57 (0.46-0.67)
**Total score CPS****	**0.96 (0.94-0.97)**	**0.63 (0.53-0.71)**	**0.70 (0.62-0.77)**	**0.33 (0.19-0.46)**	**0.43 (0.30-0.55)**
Rescue*	0.78 (0.66-0.89)	0.23 (0.14-0.32)	0.47 (0.28-0.65)	0.59 (0.41-0.77)	0.53 (0.37-0.68)
Simple descriptive scale*	0.93 (0.91-0.96)	0.77 (0.68-0.87)	0.59 (0.49-0.69)	0.76 (0.69-0.82)	0.37 (0.25-0.49)
Simple numeric scale*	0.98 (0.97-0.99)	0.82 (0.73-0.90)	0.66 (0.54-0.77)	0.84 (0.78-0.89)	0.33 (0.22-0.45)
Visual analog scale**	0.98 (0.97-0.98)	0.83 (0.77-0.87)	0.64 (0.54-0.72)	0.79 (0.72-0.84)	0.22 (0.07-0.36)

UHAPS: Unesp-Botucatu horse acute pain scale; CPS: Composite Orthopedic Pain Scale. Reference evaluator was the one with the highest intra-observer reliability.
