# Supplementary material for: A critical evaluation for validation of composite and unidimensional postoperative pain scales in horses
Source: PLoS One. 2021 Aug 5;16(8):e0255618. doi: 10.1371/journal.pone.0255618 (PMC8341545; doi:10.1371/journal.pone.0255618)
Supplement: S5 Table — (PDF) [file pone.0255618.s005.pdf]

S5 Table 5. Median (range) scores of the UHAPS, CPS and unidimensional scales in the perioperative period in horses.

	M0	M1	M2	M3
**UHAPS**				
Reference evaluator	1^{b} (0 – 8)	3^{a} (0 – 8)	3^{a} (0 – 9)	3^{ab} (0 – 8)
Lead investigator	2^{b} (0 – 6)	4^{a} (0 – 8)	3.5^{a} (0 – 9)	3^{ab} (0 – 10)
Equine internist	0^{b} (0 – 6)	1.5^{a} (0 – 7)	2^{a} (0 – 7)	1^{ab} (0 – 7)
Anesthesiologist	1^{b} (0 – 7)	2^{a} (0 – 10)	2^{ab} (0 – 7)	2^{ab} (0 – 10)
Veterinary student	1^{b} (0 – 6)	2^{a} (0 – 5)	1.5^{ab} (0 – 5)	2^{ab} (0 – 7)
Veterinary technician	1^{b} (0 – 7)	3^{a} (0 – 11)	3^{a} (0 – 8)	3^{ab} (0 – 9)
**CPS**				
Reference evaluator	3^{b} (0 – 10)	5^{ab} (0 – 15)	5.5^{a} (0 – 18)	4^{ab} (0 – 17)
Lead investigator	3^{b} (0 – 11)	5^{ab} (0 – 13)	5.5^{a} (0 – 17)	4^{ab} (0 – 14)
Equine internist	2 (0 – 8)	3 (0 – 9)	3 (0 – 13)	3 (0 – 13)
Anesthesiologist	2^{b} (0 – 11)	$3{,}5^{a}$ (0 – 11)	4^{ab} (0 – 16)	4^{ab} (0 – 14)
Veterinary student	3 (0 – 7)	4 (0 – 9)	3 (0 – 13)	4 (0 – 12)
Veterinary technician	4^{b} (0 – 14)	7^{a} (1 – 16)	7^{ab} (0 – 17)	6^{ab} (0 – 18)
**Rescue indication**[1]				
Reference evaluator	0 (0 – 1)	1 (0 – 1)	1 (0 – 1)	1 (0 – 1)
Lead investigator	0 (0 – 1)	1 (0 – 1)	1 (0 – 1)	1 (0 – 1)
Equine internist	0 (0 – 1)	1 (0 – 1)	1 (0 – 1)	1 (0 – 1)
Anesthesiologist	0 (0 – 1)	1 (0 – 1)	1 (0 – 1)	1 (0 – 1)
Veterinary student	0 (0 – 1)	1 (0 – 1)	1 (0 – 1)	1 (0 – 1)
Veterinary technician	0^{b} (0 – 1)	1^{a} (0 – 1)	1^{a} (0 – 1)	1^{ab} (0 – 1)
**Simple descriptive**				
Reference evaluator	$0^{\mathbf{b}}$ (0 – 4)	1^{a} (0 – 4)	1^{a} (0 – 5)	1^{ab} (0 – 5)
Lead investigator	0^{b} (0 – 4)	1^{a} (0 – 4)	1^{a} (0 – 4)	1^{ab} (0 – 4)

Equine internist	0^{b} (0 – 4)	1^{a} (0 – 3)	1^{a} (0 – 4)	1^{ab} (0 – 4)
Anesthesiologist	0.5^{b} (0 – 3)	1^{a} (0 – 3)	1^{a} (0 – 3)	1^{ab} (0 – 5)
Veterinary student	0 (0 – 1)	0 (0 – 2)	0 (0 – 2)	0 (0 – 6)
Veterinary technician	0^{b} (0 – 4)	1^{a} (0 – 4)	1^{a} (0 – 5)	1^{ab} (0 – 4)
**Simple numeric**				
Reference evaluator	0^{b} (0 – 8)	2.5^{a} (0 – 9)	2^{a} (0 – 9)	1^{ab} (0 – 10)
Lead investigator	0^{b} (0 – 8)	2^{a} (0 – 9)	2^{a} (0 – 9)	1^{ab} (0 – 9)
Equine internist	0^{b} (0 – 8)	2^{a} (0 – 8)	1^{a} (0 – 9)	1b (0 – 9)
Anesthesiologist	0.5^{b} (0 – 6)	2^{a} (0 – 6)	2^{ab} (0 – 7)	2^{ab} (0 – 10)
Veterinary student	0.5 (0 – 3)	1 (0 – 3)	0 (0 – 3)	1 (0 – 10)
Veterinary technician	0^{b} (0 – 8)	2.5^{a} (0 – 9)	2.5^{a} (0 – 10)	2^{ab} (0 – 9)
**Visual analog scale**				
Reference evaluator	0^{b} (0 – 8.8)	2.7^{a} (0 – 10)	2.1^{a} (0 – 10)	1.1^{a} (0 -10)
Lead investigator	0^{b} (0 - 8.5)	2.3^{a} (0 - 9.2)	2^{a} (0 – 9)	1.1^{ab} (0 - 9.6)
Equine internist	0^{b} (0 - 7.8)	1.5^{a} (0 - 7.6)	1^{a} (0 - 8.8)	1^{ab} (0 - 9.4)
Anesthesiologist	0.6^{b} (0 - 5.8)	1.8^{a} (0 – 6)	1.9^{a} (0 - 6.1)	1.6^{ab} (0 – 10)
Veterinary student	0.5 (0 – 3)	1 (0 – 3)	0 (0 – 3)	1 (0 – 10)
Veterinary technician	0.5^{b} (0 – 8)	2.25^{a} (0.2 - 8.5)	1.95^{a} (0.2 - 9.1)	1.35^{a} (0 - 9.3)

UHAPS: Unesp-Botucatu horse acute pain scale; CPS: Composite Orthopedic Pain Scale. Total scores were compared over time (M0 *vs* M1 *vs* M2 *vs* M3) by Friedman test, with p-value corrected by Bonferroni. Different letters express differences over time (a>b). [1] For the dichotomic item 'rescue indication' (0 = no indication of rescue analgesia; 1 = indication of rescue analgesia), the logistical regression using the Tukey test as a *post-hoc* test was applied. Results were the same for all evaluators; however, differences were significant only for Veterinary technician. M0 – before surgery; M1 – up to 4 hours after surgery; M2 - up to 3 hours after analgesic treatment; M3 - 24 hours after surgery.
