# Supplementary material for: A critical evaluation for validation of composite and unidimensional postoperative pain scales in horses
Source: PLoS One. 2021 Aug 5;16(8):e0255618. doi: 10.1371/journal.pone.0255618 (PMC8341545; doi:10.1371/journal.pone.0255618)
Supplement: Attachment — Submitted filename: Response to Reviewers.docx [file pone.0255618.s007.docx]

Additional Editor Comments (if provided):

The manuscript was revised by experts and it requires a major revision before it can be accepted for publication. Please carefully check the comments made by reviewer #2 and revise the manuscript.

*Answer: The authors appreciate the editor and the reviewers for taking the time and effort to review this manuscript. All corrections were performed according to the editor´s and reviewers' suggestions, as described below*

Journal Requirements:

When submitting your revision, we need you to address these additional requirements.

1) Please ensure that your manuscript meets PLOS ONE's style requirements, including those for file naming. The PLOS ONE style templates can be found at

<https://journals.plos.org/plosone/s/file?id=wjVg/PLOSOne_formatting_sample_main_body.pdf> and

<https://journals.plos.org/plosone/s/file?id=ba62/PLOSOne_formatting_sample_title_authors_affiliations.pdf>

*Answer: adjustments have been performed to meet PLOS ONE´s style requirements*

2) Thank you for stating the following in the Competing Interests section:

[The authors have declared that no competing interests exist.].

We note that you received funding from a commercial source: Narkovet Consulting®, LLC,

Please provide an amended Competing Interests Statement that explicitly states this commercial funder, along with any other relevant declarations relating to employment, consultancy, patents, products in development, marketed products, etc.

Within this Competing Interests Statement, please confirm that this does not alter your adherence to all PLOS ONE policies on sharing data and materials by including the following statement: "This does not alter our adherence to PLOS ONE policies on sharing data and materials.” (as detailed online in our guide for authors http://journals.plos.org/plosone/s/competing-interests).  If there are restrictions on sharing of data and/or materials, please state these. Please note that we cannot proceed with consideration of your article until this information has been declared.

Please include your amended Competing Interests Statement within your cover letter. We will change the online submission form on your behalf.

Please know it is PLOS ONE policy for corresponding authors to declare, on behalf of all authors, all potential competing interests for the purposes of transparency. PLOS defines a competing interest as anything that interferes with, or could reasonably be perceived as interfering with, the full and objective presentation, peer review, editorial decision-making, or publication of research or non-research articles submitted to one of the journals. Competing interests can be financial or non-financial, professional, or personal. Competing interests can arise in relationship to an organization or another person. Please follow this link to our website for more details on competing interests: <http://journals.plos.org/plosone/s/competing-interests>

*Answer: The Competing Interests Statement has been included in the cover letter and manuscript.*

3) Please include captions for your Supporting Information files at the end of your manuscript, and update any in-text citations to match accordingly. Please see our Supporting Information guidelines for more information: <http://journals.plos.org/plosone/s/supporting-information>.

*Answer: supporting Information file captions were included, and citations were updated in-text*

4) Your ethics statement should only appear in the Methods section of your manuscript. If your ethics statement is written in any section besides the Methods, please delete it from any other section.

*Answer: the ethics statement appears only in Methods section now.*

[Note: HTML markup is below. Please do not edit.]

Reviewers' comments:

Reviewer's Responses to Questions

**Comments to the Author**

1. Is the manuscript technically sound, and do the data support the conclusions?

The manuscript must describe a technically sound piece of scientific research with data that supports the conclusions. Experiments must have been conducted rigorously, with appropriate controls, replication, and sample sizes. The conclusions must be drawn appropriately based on the data presented.

Reviewer #1: Yes

Reviewer #2: Partly

2. Has the statistical analysis been performed appropriately and rigorously?

Reviewer #1: Yes

Reviewer #2: Yes

3. Have the authors made all data underlying the findings in their manuscript fully available?

The [PLOS Data policy](http://www.plosone.org/static/policies.action#sharing) requires authors to make all data underlying the findings described in their manuscript fully available without restriction, with rare exception (please refer to the Data Availability Statement in the manuscript PDF file). The data should be provided as part of the manuscript or its supporting information, or deposited to a public repository. For example, in addition to summary statistics, the data points behind means, medians and variance measures should be available. If there are restrictions on publicly sharing data—e.g. participant privacy or use of data from a third party—those must be specified.

Reviewer #1: Yes

Reviewer #2: Yes

4. Is the manuscript presented in an intelligible fashion and written in standard English?

PLOS ONE does not copyedit accepted manuscripts, so the language in submitted articles must be clear, correct, and unambiguous. Any typographical or grammatical errors should be corrected at revision, so please note any specific errors here.

Reviewer #1: Yes

Reviewer #2: Yes

5. Review Comments to the Author

Please use the space provided to explain your answers to the questions above. You may also include additional comments for the author, including concerns about dual publication, research ethics, or publication ethics. (Please upload your review as an attachment if it exceeds 20,000 characters)

Reviewer #1: The manuscript presents excellent results from the assessment of different pain scales (composite and unidimensional) in the postoperative period of horses.

The study presents a robust statistical evaluation and analyzes criteria that are important to understand the advantages and limitations of the different types of scale.
I suggest adding the p values for the Spearman correlation analysis in tables 3 and 6.

*Answer: the authors appreciate your time and effort in reviewing this manuscript and your positive comments.*

*The p values for the Spearman correlation analysis were included in tables 3 and 6 as required.*

Reviewer #2: The authors are applauded for their efforts to analyze and report this complicated analysis of a large data set. The topic is important to equine medicine/surgery, the experimental design is well-explained, and the information is likely important in our continued search for the best possible way to assess/measure pain in equine patients. It is always challenging to conduct this type of pain assessment in a non-standardized population of clinical patients. I think the authors do a good job in trying to manage the extraneous variables and diminish bias. The manuscript suffers, however, from a lack of focus and clarity. The manuscript is difficult to interpret because of the wide variety of analyses, acronyms, and competing/conflicting statements.

*Answer: the authors appreciate your time and effort in reviewing this manuscript and your constructive comments. We did our best to improve clarity. Although an abbreviation list was already available in the previous version, we excluded the acronyms in the text to provide an easier reading.*

A table of acronyms should be included for reference. A similar table with concise definitions of terminology such as reliability, repeatability, sensitivity, specificity, consistency, validity, etc would be helpful. I realize that descriptors for most terms are included in Table 1 in conjunction with a description of the statistical tests which were utilized, but the definitions are not easily interpreted by scientists who may not be experts in this type of experimental design. I strongly recommend that the manuscript be written with language that makes the information more broadly accessible without sacrificing the precision that is important for interpretation of results.

*Answer: concise definitions of terminology and the rationale behind the methods used for statistical analysis has been included as a new column in Table 1. Results are better explained in lines 301-303, 339-348, 351-354 and, 392-395 of the clean version of the manuscript. Some comments have also been included in Discussion (lines 499-511,514-515, and 530-532 of the clean version of the manuscript). We hope the manuscript is more comprehensible and easier to follow.*

The stated objective for the study is to compare results obtained with select unidimensional or composite pain scales by evaluators with variable backgrounds. I don't think that the authors can draw any meaningful conclusions about the experience of evaluators based on only 1 of each type of individual. A much larger number of individuals (e.g. many students, many technicians, many surgeons, many internists) would be required to make conclusions regarding whether the experience of the individual performing the assessment has a significant impact on the assessment.

*Answer: the authors agree with your comment. It would be necessary at least six individuals of each category to draw any conclusion about the results of the observers. All points regarding this topic were excluded from Discussion.*

It was interesting that the need for rescue analgesia appeared to have the most intra-observer variability. This type of assessment may depend on many factors other than the perceived level of pain. Some individuals believe that it is reasonable for post-operative patients to experience moderate pain while other individuals believe all pain should be minimized or treated. For example, some individuals may believe that pain should be treated with a VAS of 2 and others may believe that pain should not be treated unless there was a VAS of 8. These are philosophical differences that were not addressed in the discussion. I'm not sure that the need for rescue analgesia is appropriate to be included in the analysis in the same way as the presumably more objective scoring systems. (This doesn't mean that it shouldn't be included in the manuscript but perhaps it should be handled in a different way or at least discussed.)

*Answer: Thank you for your comment. This was included in Discussion (lines 465-469 of the clean version of the manuscript). According to your suggestion, rescue analgesia results were excluded from Tables 2 and 4 of the main text, as the same information is provided in Supporting Information (S3 and S4 Tables).*
